# Mitochondrial Genetics Reinforces Multiple Layers of Interaction in Alzheimer’s Disease

**DOI:** 10.3390/biomedicines10040880

**Published:** 2022-04-12

**Authors:** Giovanna Chaves Cavalcante, Leonardo Miranda Brito, Ana Paula Schaan, Ândrea Ribeiro-dos-Santos, Gilderlanio Santana de Araújo, on behalf of Alzheimer’s Disease Neuroimaging Initiative

**Affiliations:** Laboratory of Human and Medical Genetics, Graduate Program in Genetics and Molecular Biology, Institute of Biological Sciences, Federal University of Pará, Belém 66075-110, Brazil; giovanna.cavalcante@icb.ufpa.br (G.C.C.); leonardo.brito@itec.ufpa.br (L.M.B.); ana.schaan@icb.ufpa.br (A.P.S.); akely@ufpa.br (Â.R.-d.-S.)

**Keywords:** differential expression, epistasis, mtDNA, Alzheimer’s Disease, cerebrospinal fluid, TAU, PTAU

## Abstract

**Simple Summary:**

Nuclear DNA remains the main source of genome-wide loci association in neurodegenerative diseases, only partially accounting for the heritability of Alzheimer’s Disease (AD). In this context, mitochondrial DNA (mtDNA) is gaining more attention. Here, we investigated mitochondrial genes and genetic variants that may influence mild cognitive impairment and AD, through an integrative analysis including both differential gene expression and mitochondrial genome-wide epistasis analysis. Our results highlight important layers of interactions involving mitochondrial genetics and suggest specific molecular alterations as potential biomarkers for AD.

**Abstract:**

Nuclear DNA has been the main source of genome-wide loci association in neurodegenerative diseases, only partially accounting for the heritability of Alzheimer’s Disease (AD). In this context, mitochondrial DNA (mtDNA) is gaining more attention. Here, we investigated mitochondrial genes and genetic variants that may influence mild cognitive impairment and AD, through an integrative analysis including differential gene expression and mitochondrial genome-wide epistasis. We assessed the expression of mitochondrial genes in different brain tissues from two public RNA-Seq databases (GEO and GTEx). Then, we analyzed mtDNA from the ADNI Cohort and investigated epistasis regarding mitochondrial variants and levels of A$\beta_{1-42}$, TAU, and Phosphorylated TAU (PTAU) from cognitively healthy controls, and both mild cognitive impairment (MCI) and AD cases. We identified multiple differentially expressed mitochondrial genes in the comparisons between cognitively healthy individuals and AD patients. We also found increased protein levels in MCI and AD patients when compared to healthy controls, as well as novel candidate networks of mtDNA epistasis, which included variants in all mitochondrially-encoded oxidative phosphorylation complexes, 12S rRNA and MT-DLOOP. Our results highlight layers of potential interactions involving mitochondrial genetics and suggest specific molecular alterations as potential biomarkers for AD.

## 1. Introduction

Alzheimer’s Disease (AD) is a progressive neurodegenerative condition with a complex origin that leads to a myriad of symptoms, such as severe memory loss, confusion, multiple cognitive deficiencies and personality changes. AD is the most common cause of dementia, responsible for 60–80% of cases worldwide [1]. AD progression is slow—gradually worsening over years or even decades—and a final diagnosis is commonly reached in moderate to severe stages due to unspecific traits of cognitive symptoms during early stages [2]. AD occurs in a late-onset sporadic form (at least 65 years of age, representing 95% of the cases) or in an early-onset familial form (under 65 years of age, representing 5% of the cases) [1,3]. According to the 2020 report by the Alzheimer’s Association, most patients live 4–8 years after diagnosis and deaths officially related to AD increased by 146.2% from 2000 to 2018 [4]. This report also highlights that molecular changes may begin over 20 years before AD symptoms arise, which represents a window of opportunity for early interventions during disease development and progression.

Notably, AD presents heterogeneity regarding etiology, symptomatology, age of onset and rates of progression, but the mechanisms involved in this diversity of disease presentation are still not fully comprehended [5]. The heterogeneous nature of AD also poses an obstacle towards developing standard treatments [6]. Currently, there are treatments that may help to slow some of the symptoms, but there is no cure for the disease [3]. Thus, genetic, epigenetic and environmental factors have been explored in an effort to elucidate the neurodegenerative process through a holistic perspective [7,8,9].

Histopathologically, AD cases show an accumulation of intraneuronal hyperphosphorylated TAU protein (PTAU) and extracellular plaques of amyloid-β (Aβ or AB) in the brain [1]. These molecular changes are related to different genetic and epigenetic factors, including those involved in mitochondrial activity, particularly in energy generation. Indeed, mitochondria have been increasingly associated with neurological diseases, including AD, although it is still unclear whether mitochondrial alterations are a primary or a secondary event in this disease [10]. Mitochondrial alterations have also been found in mild cognitive impairment (MCI), a state that frequently progresses to AD [11,12].

In the last few decades, a link between the mitochondrial DNA (mtDNA) and MCI and AD has been strongly suggested, but variants of interest in regions of mtDNA origin and their biological functions remain poorly understood. Additionally, epistasis—the dependent effect that multiple genetic variants have on a trait, i.e., the occurrence of at least two genetic variants with a different effect on a specific trait [13]—has been pointed out as an important and neglected factor in the heritability of AD [14]. In this context, recent studies have highlighted the need for investigating rare and/or overlooked variants that may interact and influence the risk of neurodegeneration in AD development [15,16].

Therefore, in this study, we aimed to identify mitochondrial genes and genetic variants that may have a role in the development of AD, by performing an integrative data analysis from independent sources. First, we conducted a prospective study on the differential expression of mitochondrial genes in various brain tissues. Then, we analyzed epistatic mtDNA genetic associations with cerebrospinal fluid (CSF) levels of $A\beta_{1-42}$, TAU and PTAU based on public datasets, as well as the private cohort of the Alzheimer’s Disease Neuroimaging Initiative (ADNI) [17,18]. Here, we show differential expression between mtDNA genes and pseudogenes in AD patients as well as candidate mtDNA genotype interactions that may influence TAU and PTAU levels in CSF from MCI and AD patients.

## 2. Materials and Methods

Our methodology consisted of different approaches to analyzing gene regulation and genomics from mtDNA data. First, differential expression (DE) analyses of mitochondrial genes were performed. DE analyses were based on RNA-seq data from brain tissues sampled from healthy young adults (HYA), healthy elderly adults (HEA) and Alzheimer’s Disease cases (AD). Next, we investigated epistasis of mitochondrial genome variants genotyped for AD, MCI and healthy individuals from the ADNI Cohort. Then, genome-wide association analyses were performed based on mitochondrial data. SNP-SNP interaction analyses were performed for mtDNA variants in a quantitative design to investigate candidate associations with cerebrospinal levels of $A\beta_{1-42}$, TAU and PTAU.

### 2.1. RNA-seq Transcriptome Data from Brain Tissues

First, we processed two available RNA-seq experiments found in the Gene Expression Omnibus (GEO) for brain tissues. Transcriptome data were generated for lateral temporal lobe and fusiform gyrus tissues by third parties and both experiments are stored in GEO under accession numbers GSE104704 and GSE125583, respectively. Sample numbers and age ranges of the individuals included in these experiments are presented in Table 1.

In addition to the aforementioned brain tissues, we extracted RNA-seq data from the Genotype Tissue Expression database (GTEx, v.8), which provides genome-wide data and a summary of expression quantitative trait loci analysis for a large set of tissue donors [18]. This allowed us to extract transcriptome data from 54 tissues, from 948 donors and a total of 17,382 samples. Around 50% of the donors presented ages ranging from 60 to 70 years old. Out of GTEx large data, we extracted expression data for 13 different brain tissues. For further analyses, these data were used to explore gene expression patterns in healthy brains, which allowed us to investigate how different genes and pseudogenes are expressed at baseline.

### 2.2. ADNI Cohort Data: mtDNA Sequence Data and Biomarkers

Mitochondrial genomes were sequenced for 809 individuals according to the protocol previously described in [19]. According to [20,21], the individuals included in this cohort are mainly of mitochondrial European ancestry, mostly belonging to haplogroups H, I, J and K, widely present throughout the European continent; by protocol, individuals were clinically analyzed using medical resonance imaging, cognition measurement scores, APOE genotyping and a series of biomarkers extracted from plasma and CSF.

The ADNI longitudinal database stores CSF data from $A\beta_{1-42}$, total TAU (TTAU or TAU) and phosphorylated TAU at threonine 181 (PTAU) levels collected at baseline (N = 475) after diagnostic examinations for healthy (N = 166), MCI patients (N = 268) and AD cases (N = 41). Measurements of these biomarker levels met all quality control (QC) requirements as described in [22]. At baseline, individuals were, on average, 73 years old (+/−7.18). Of these, 253 were male and 222 were females.

### 2.3. Differential Gene Expression Analysis in Brain Tissues

In order to process the RNA-seq experiments, we downloaded *.sra* files and converted them to *.fastq* files using the SRA-Toolkit [23]. QC was assessed by FastQC and MultiQC [24,25]. Trimmomatic was performed for read cleaning and a second round of QC [26].

After QC, reads were aligned to the UCSC reference genome (University of California Santa Cruz), version hg19 (http://hgdownload.soe.ucsc.edu/downloads.html, accessed on 4 August 2021), using the STAR tool [27], and annotated following the coordinate regions of the Gencode genome (v19) (https://www.gencodegenes.org/human/release_19.html, accessed on 4 August 2021). Finally, read counting was performed using the HTseq library [28], implemented in the Python programming language, using the function scripts.count, thus generating files with the abundance of each gene per sample.

DE analyses of the three experiments were performed with the edgeR package in R version 3.5.2 [29]. In this analysis, the gene count matrix was filtered for reads with more than 10 counts. The resulting matrices were normalized, thereby converting expression values to a scale that allows adequate data comparisons. Lastly, we adjusted the gene counts to a generalized negative binomial log-linear model and controlled for multiple testing with False Discovery Rate (FDR ≤ 0.05).

### 2.4. Gene–Gene Interaction Network Analysis

GeneMANIA (www.genemania.org, accessed on 4 August 2021) is a user-friendly web tool for generating hypotheses about gene function and gene prioritization support [30]. GeneMANIA finds similar genes using functional genomics, transcriptomics and proteomics data given a seed gene list. The tool receives as input a list of genes to build a gene–gene interaction network, which is based on extensive biological datasets for functional similarity analysis, co-expression or even interactions at protein level. Here, we employed GeneMANIA to analyze whether seed mtDNA genes and their interactions could be involved in the development of AD or interact with other nuclear genes related to neurogenerative processes.

### 2.5. Epistasis between mtDNA Variants with an Impact on CSF Biomarker Levels

Epistasis analyses were performed with multiple linear regression for quantitative traits. The linear model is based on the allele dosage for each mtDNA variant, *A* and *B*, and fits the following model, in which the interaction test is based on the $b_{3}$ coefficient:

(1) $$Y=b_{0}+b_{1}\cdot A+b_{2}\cdot B+b_{3}\cdot AB+e$$

The mtDNA variants were filtered for minor allele frequency (MAF) greater than 0.01 and 99% genotyping rate; monomorfic SNPs were removed. MAF threshold was used to exclude rare SNPs from our analysis. Then, epistasis analyses were performed in the context of AD considering the ADNI groups (AD, MCI and healthy subjects, N = 475) and association analyses with AB, PTAU and TAU levels. Next, we conducted a mitochondrial genome-wide epistasis analysis using a linear regression model for quantitative trait analysis association. The linear regression was implemented in PLINK software and was executed with the *–epistasis* command, release v1.9 [31]. Association hits with a *p*-value ≤ 5.0 $\times 10^{-5}$ were considered statistically significant.

## 3. Results

### 3.1. Screening for Differential Expression of mtDNA Genes and Pseudogenes

Three comparative analyses of gene expression were performed for the two datasets: HYA vs. AD (GSE104704), HEA vs. AD (GSE104704) and HEA vs. AD (GSE125583). When comparing HYA vs. AD (lateral temporal lobe), 12 genes were found to be DE, while three were DE in HEA vs. AD comparisons (lateral temporal lobe and fusiform gyrus). Considering them individually, there were 15 DE genes in total: five tRNA genes, eight pseudogenes and two isoforms.

As seen in Table 2, the following genes were differentially expressed in the investigated tissues: *MT-TL1* (Mitochondrially Encoded TRNA-Leu 1), *MT-TV* (Mitochondrially Encoded TRNA-Val), *MT-TM* (Mitochondrially Encoded TRNA-Met), *MT-TH* (Mitochondrially Encoded TRNA-His), *MT-TS2* (Mitochondrially Encoded TRNA-Ser 2), *MTND2P28* (MT-ND2 pseudogene 28), *MTND1P23* (MT-ND1 pseudogene 23), *MTND1P20* (MT-ND1 pseudogene 20), *MTND1P21* (MT-ND1 pseudogene 21), *MTND5P11* (MT-ND5 pseudogene 11), *MTND4P9* (MT-ND4 pseudogene 9), *MTND2P12* (MT-ND2 pseudogene 2), *MTRNR2L1* (MT-RNR2 like 1), *MTND6P3* (MT-ND6 pseudogene 3) and *MTRNR2L2* (MT-RNR2 like 2).

Notably, *MTRNR2L1* and *MTND1P23* appeared in the results of more than one analysis: *MTRNR2L1* was DE in two (with downregulated expression in both lateral temporal lobe tissues) and MTND1P23 was DE in all three analyses (with upregulated expression in both lateral temporal lobe tissues, while downregulated in the fusiform gyrus tissue). This pattern suggests a tissue-specific regulation of such genes in AD. The other DE genes presented individual patterns in the investigated tissues, i.e., only appeared once.

**Figure 1:**
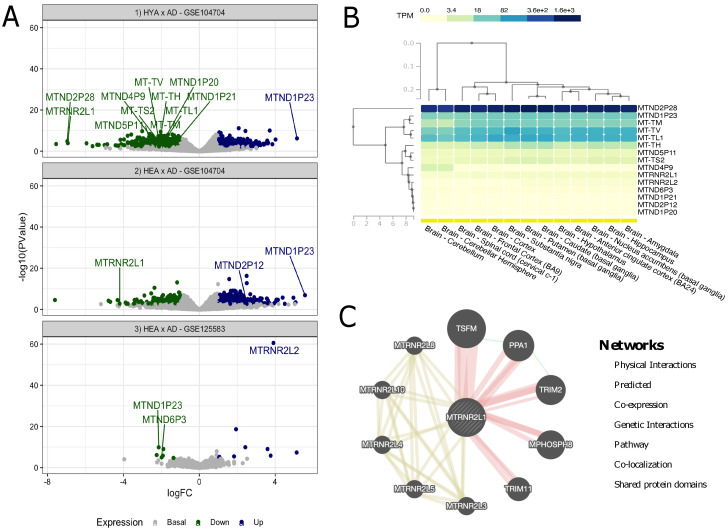
Analyses of the GEO and GTEx samples. (**A**) Volcano plot of DE analysis for Alzheimer’s disease; the marked dots are differentially expressed mitochondrial DNA genes, while all others are nuclear genes; (**B**) GTEx biclustering for mtDNA genes in 13 brain tissues; (**C**) gene–gene interaction network of MTRNR2L1.

In addition to Table 2, we plotted the results from the previous analysis for all three experiments (HYA vs. AD in lateral temporal lobe, HEA vs. AD in lateral temporal lobe and HEA vs. AD in fusiform gyrus), as a clear representation of the DE genes, as shown in Figure 1A. To gain insight into the expression of such genes, we performed a comparative expression analysis of all 15 genes in 13 healthy brain tissues with data extracted from the GTEx portal, which did not include fusiform gyrus or lateral temporal lobe tissues specifically (Figure 1B). From these results, interaction networks were searched for each gene in GeneMANIA, but were only found for *MTRNR2L1* (Figure 1C).

As seen in Figure 1B, out of the 15 genes, five were highly overexpressed in all of the investigated tissues (*MTND2P28*, *MTND1P23*, *MT-TM*, *MT-TV* and *MT-TL1*), with two exceptions (*MT-TM* was mildly overexpressed in the cerebellum and cerebellar hemisphere). It should be noted that *MTND2P28* presented much higher levels of expression than others. Other mildly overexpressed genes in all tissues included *MT-TH*, *MTND5P11* and *MT-TS2*. All other genes showed similar underexpression patterns and, to some extent, were grouped together. As for the tissues, it is noteworthy that, based on gene expression patterns, the group formed by the amygdala and hippocampus was similar to nucleus accumbens (basal ganglia) and that this cluster was also similar to another, formed by the anterior cingulate cortex and hypothalamus; these tissues also presented great similarity with the caudate and putamen, both basal ganglia regions and, to a lesser extent, substantia nigra (also part of the basal ganglia). Cortex and frontal cortex were also grouped together, and these tissues were close to the spinal cord. Lastly, the cerebellum and cerebellar hemisphere formed a cluster that, although still similar, was the most separated from the others.

In the interaction network for *MTRNR2L1* shown in Figure 1C, we found 10 strongly linked genes, of which five seemed to present physical interactions (*MPHOSPH8*, *PPA1*, *TRIM2*, *TRIM11* and *TSFM*) and the other five shared protein domains not only with *MTRNR2L1* but also with each other (*MTRNR2L3*, *MTRNR2L4*, *MTRNR2L5*, *MTRNR2L8* and *MTRNR2L10*). In addition, *PPA1* (Inorganic Pyrophosphatase 1) presented genetic interactions with *TSFM* (Ts Translation Elongation Factor, Mitochondrial) and *TRIM2* (Tripartite Motif Containing 2).

### 3.2. Epistasis between mtDNA and CSF PTAU and TAU Levels

Then, we hypothesized that some patterns of differential expression observed in the previous analysis could result from the effects caused by the presence of mtDNA variants, so we searched the ADNI data for epistasis between mtDNA genes related to $A\beta_{1-42}$, PTAU and TAU levels extracted from CSF. Interestingly, linear regression results revealed epistatic interactions among mtDNA variants and PTAU and TAU levels, but not for A$\beta_{1-42}$.

**Figure 2:**
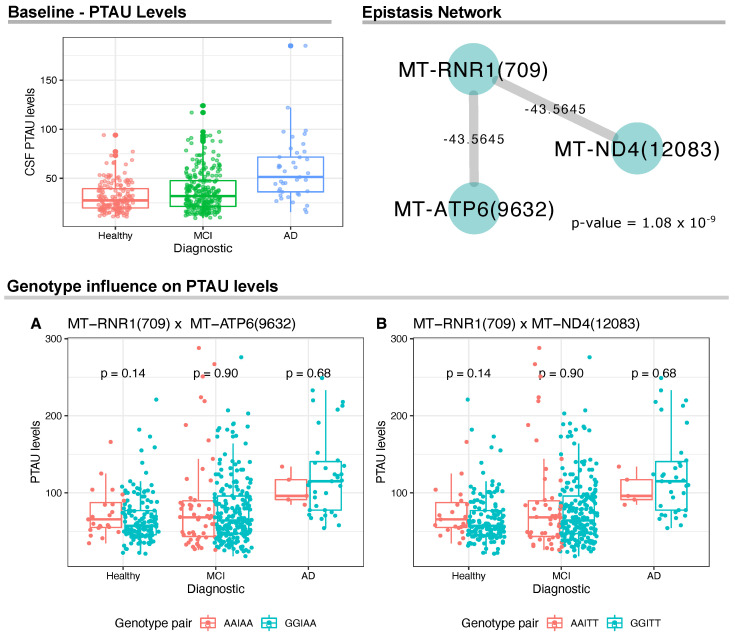
Analyses of the CSF PTAU levels and mtDNA variants. On the top left, we show the CSF PTAU levels at baseline for the ADNI cohort. On the top right, we show the epistasis network with statistical significance between mtDNA variants and PTAU CSF levels. The network was made from linear model analysis (*p*-value ≤ 1.0 $\times 10^{-9}$). Wilcoxon test results and distribution of mtDNA genotype pairs in relation to PTAU levels.

For PTAU levels, we identified a significant gene network between *MT-RNR1*(709) and two variants, namely *MT-ATP6*(9632) and *MT-ND4*(12083) (Figure 2); however, we did not find statistically significant differences between groups of genotype pairs when analyzing PTAU levels within each sample group (see Figure 2A,B).

**Figure 3:**
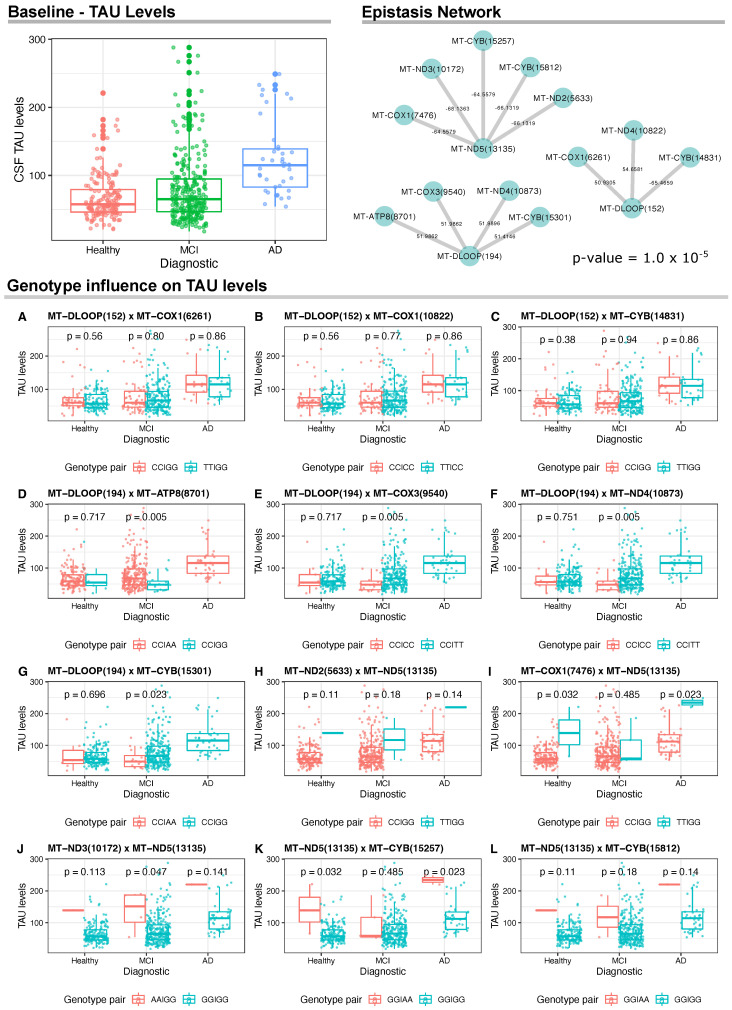
Analyses of the CSF TAU levels and mtDNA variants. On the top left, we show the CSF TAU levels at baseline for the ADNI Cohort. On the top right, we show the epistasis interactions between mtDNA variants and TAU levels that reached a linear model (*p*-value ≤ 1.0 $\times 10^{-5}$). Wilcoxon test results and distribution of mtDNA genotype pairs in relation to TAU levels.

As for CSF TAU levels, AD presented higher levels when compared to healthy and MCI groups. In addition, there were three networks of gene interactions, which involved the mitochondrial regions *MT-ND5*(13135), MT-DLOOP(194) and MT-DLOOP(152) (Figure 3). For *MT-ND5*(13135), we found negative epistasis with five genes: *MT-COX1*(7476), *MT-ND3*(10172), *MT-CYB*(15257), *MT-CYB*(15812), *MT-ND5*(5633). Results for MT-DLOOP(194) returned positive epistasis with four genes, namely *MT-ATP6*(8701), *MT-COX3*(9540), *MT-ND4*(10873), *MT-CYB*(15301). Lastly, two positive and one negative epistatic interactions were identified for MT-DLOOP(152). The positive interactions involved *MT-COX1*(6261) and *MT-ND4*(10822) and the negative interaction involved *MT-CYB*(14831).

We also carried out a sex-specific analysis to investigate epistasis between males and females. For PTAU levels, the entire epistasis network was replicated only for female samples, resulting in both interactions MT-RNR1(709) × MT-ATP6(9632) and MT-RNR1(709) × MT-ND4(12083) (*p*-value = 7.9 $\times 10^{-9}$). Considering TAU levels, we did not observe specific effects for females. However, epistasis was observed specifically in men between the pair of variants MT-DLOOP(239) × MT-ND2(4917) (*p*-value = 8.5 $\times 10^{-5}$).

When analyzing CSF TAU levels among sample groups in relation to the genotypes of these three variants, we found statistically significant associations for MT-DLOOP(194) and *MT-ND5*(13135), but not for MT-DLOOP(152) (Figure 3A–C). For instance, for MT-DLOOP(194), we found that individuals with MCI carrying CC/AA genotypes in this variant and MT-ATP8(8701), respectively, had significantly higher CSF TAU levels when compared to CC/GG genotype carriers (Figure 3D). Data for CC/GG individuals with AD were not available for comparison. This same pattern was observed for CC/TT carriers when analyzing the MT-DLOOP(194) ×*MT-COX3*(9540) and MT-DLOOP(194) × *MT-ND4*(10873) epistatic interactions (Figure 3E,F). Lastly, for MT-DLOOP(194), we found significantly higher levels of CSF TAU in MCI patients with CC/GG genotypes for MT-DLOOP(194) and *MT-CYB*(15301) (Figure 3G).

Regarding the epistatic interactions with *MT-ND5*(13135), there was no statistical significance in the comparison of carriers of *MT-ND2*(5633) and *MT-ND5*(13135) (Figure 3H), but we found higher levels of CSF TAU in healthy and AD individuals with the TT/GG genotype for *MT-COX1*(7476) and *MT-ND5*(13135) in comparison to the analyzed pair for these variants (Figure 3I). Carriers of GG/GG for *MT-ND3*(10172) and *MT-ND5*(13135) with MCI had lower TAU levels and this same pattern was also seen for carriers of GG/GG in *MT-ND5*(13135) and *MT-CYB*(15257) among healthy and AD individuals (Figure 3J,K). No statistical significance was observed in the comparisons for *MT-ND5*(13135) and *MT-CYB*(15812) (Figure 3L).

## 4. Discussion

Mitochondria are organelles crucial for cellular balance and function, being responsible for several pathways and standing out for their role in energy generation through aerobic respiration—the tricarboxylic acid (TCA) cycle and, particularly, oxidative phosphorylation (OXPHOS) [32]. This function is so vital that the human mitochondrial genome (mtgenome or mitogenome) is highly specialized: it contains only 37 genes: 13 encoding proteins, all of which are subunits of the electron transport chain (ETC) complexes where OXPHOS occurs, and the remaining are part of the RNA machinery (22 tRNAs and 2 rRNAs), in addition to non-coding regions, so that all other mitochondrial proteins (over 1000) are encoded by the nucleus [33]. Hence, alterations in these mitochondrial or nuclear genes lead to mitochondrial dysfunction, affecting cellular homeostasis to promote the development of diseases. In this context, neurons would be specially affected, as they consume a large amount of energy, thus being especially dependent on mitochondrial metabolism for energy generation and other functions, such as neurotransmission and neuroplasticity [34,35].

In 2004, a mitochondrial cascade hypothesis was first proposed for late-onset sporadic AD considering that: (i) mitochondrial function decreases with age; (ii) mtDNA damage may be triggered by excessive reactive oxygen species (ROS), which are produced during OXPHOS; and (iii) alterations in mtDNA might reduce ETC efficiency from a basal inherited level [36]. Once the mitochondrial dysfunction reaches a certain threshold, a compensatory response would be induced and some histopathological characteristics of AD would arise as a consequence of this response [37]. Although AD is considered a multifactorial disease, this hypothesis stands out for emphasizing the importance of mtDNA to the development of this type of AD, placing mitochondria in a central position in this process. In addition, as genomic ancestry may play important roles in the development of different diseases, multiple mitochondrial haplogroups have been related to AD in independent studies [38,39].

Notably, in AD, atrophy may occur in the different areas of the brain, but atrophy of the hippocampus (located in the medial temporal lobe) has been traditionally considered a core feature of the disease [40]. In fact, researchers have observed an early divergence of the AD brain model from the normal aging trajectory in the hippocampus, lateral ventricles and amygdala [41]. Interestingly, it has also been demonstrated that volume reduction in AD may start in the medial temporal lobes (although not necessarily the hippocampus) and in the fusiform gyrus at least three years prior to AD progression, spreading to the other lobes before diagnosis [42]. Recently, a study highlighted differences across typical and atypical AD phenotypes in TAU accumulation and atrophy regions, including the lateral temporal lobe [43]. Therefore, the heterogeneous course of this disease should be further explored.

Here, we found DE mitochondrial genes when analyzing RNA-seq data from AD lateral temporal lobe and fusiform gyrus tissues. The lateral temporal lobe (or the lateral surface of the temporal lobe), delimited by superior and inferior temporal sulci and composed of three gyri (superior, middle and inferior temporal gyrus), is responsible for different visual and auditory functions, such as facial recognition, language comprehension and hearing [44,45]. The fusiform gyrus (or occipitotemporal gyrus) is located in the inferior region of the temporal and occipital lobes, being associated with high-level vision functions such as the recognition of faces, bodies and objects, as well as reading [46,47]. In AD, face–name memory—the ability to recognize faces and recall names—is markedly impaired, which could be related to degeneration in such brain regions [48]. This, in turn, could be due to molecular alterations such as genetic mutations and different gene expression levels.

Among the DE genes in the studied tissues, five are transfer RNA (tRNA) genes (*MT-TH*, *MT-TL1*, *MT-TM*, *MT-TS2* and *MT-TV*), which are crucial parts of the mitochondrial translational machinery [49]. Considering that the presence of mutations and the altered expression of mitochondrial genes may affect the functioning of mitochondria, especially in muscular and neuronal tissues, and lead to the onset and progress of different diseases [50], the alteration of tRNA genes is of great interest. The downregulated expression of tRNA genes can reduce translation efficiency and is likely to be associated with protein deficiency, which may be linked to the pathogenesis of AD.

Moreover, in our analyses, 10 of the 15 genes found to be DE in the studied tissues are classified as pseudogenes or isoforms of the following mitochondrial genes: *MT-ND1* (*MTND1P20*, *MTND1P21* and *MTND1P23*), *MT-ND2* (*MTND2P12* and *MTND2P28*), *MT-ND4* (*MTND4P9*), *MT-ND5* (*MTND5P11*), *MT-ND6* (*MTND6P3*) and *MT-RNR2* (*MTRNR2L1* and *MTRNR2L2*). To date, there are not many studies investigating these specific pseudogenes and isoforms in the global literature. In fact, to the best of our knowledge, this is the first study to explore their expression, with the exception of the *MT-RNR2* isoforms *MTRNR2L1* and *MTRNR2L2*—and no previous studies were found on the involvement of *MTRNR2L1* with any of these genes or the other genetic interactions shown here. Curiously, the isoform *MTRNR2L12* has been suggested as a potential biomarker for early AD-like dementia in individuals with Down Syndrome [51], but it was not DE in our study. Regardless, this highlights the potential role mitochondrial that isoforms might have in AD and other types of dementia.

The *MT-RNR2* gene encodes the mitochondrial 16S ribosomal RNA (rRNA), and it is also associated with the production of humanin (HN), a peptide that has been shown to be a neuroprotective factor for AD through the suppression of apoptotic cell death when discovered [52] and that, since then, has been associated with different processes and age-related diseases [53]. Humanin presents 13 isoforms encoded by nuclear *MT-RNR2*-like genes, such as *MTRNR2L1* (HN1) and *MTRNR2L2* (HN2) [54]. In the last decade, both of these isoforms have been investigated in different multifactorial diseases, although there are few studies so far.

For instance, a genome-wide association study showed a statistically significant relation of *MTRNR2L2* with the progression of Huntington’s Disease, so the role of this humanin isoform may vary among various diseases [55]. In fact, a recent review on humanin by Hazafa et al. [56] reinforced the importance of this mitochondrial-derived peptide and its isoforms in cytoprotection through the regulation of different mechanisms, including mitochondrial pathways, with a potential influence on the development and treatment of multifactorial diseases related to oxidative stress and apoptosis, which comprise neurodegenerative diseases such as AD.

Here, we found *MTRNR2L1* to be underexpressed in both lateral temporal lobe experiments and *MTRNR2L2* to be overexpressed in the fusiform gyrus of AD patients. Considering the established neuroprotective function of these humanin isoforms, these results suggest a progression of the disease in the lateral temporal lobe, but not in the fusiform gyrus. Notably, we found *MTND1P23* to be DE in all three experiments, being overexpressed in both lateral temporal lobe experiments and underexpressed in the fusiform gyrus, which suggests that *MTND1P23* might play an important role in the evolution of AD. This is particularly interesting given that all other genes in the lateral temporal lobe of AD patients in the HYA vs. AD analysis were underexpressed, possibly being protective factors for AD. Similarly, it is also possible to hypothesize, for instance, that the overexpression of *MTND2P12* may be indicative of AD progression and that the underexpression of *MTND6P3* may be a protective factor against neurodegeneration in AD. However, in the GTEx analysis with healthy brain tissues that did not include the lateral temporal lobe or fusiform gyrus, *MTND1P23* was overexpressed in all of the explored tissues. Hence, more studies are needed to clarify the possibilities involving these genes.

To further explore the influence of mitochondrial genetics for AD progression, particularly the occurrence of epistasis, we analyzed the ADNI database with whole mtgenome sequencing of AD, MCI and cognitively healthy subjects, as well as AB, PTAU and TAU levels in CSF from these groups.

Interestingly, we found differences in the CSF levels for the three proteins and epistatic interactions between multiple mtDNA variants for PTAU and TAU. For PTAU, the variant is located in the 12S rRNA gene (*MT-RNR1*) and interacts with variants in Complex I (*MT-ND4*) and Complex V (*MT-ATP6*) genes; however, these interactions do not seem to affect PTAU levels in CSF.

For TAU, two variants are located in MT-DLOOP and one variant is located in a Complex I gene (*MT-ND5*). Of the MT-DLOOP variants, one (152) interacts with variants in genes of Complexes I, III and IV (*MT-ND4*, *MT-CYB* and *MT-COX1*, respectively), but these interactions do not seem to affect TAU levels in CSF. The other MT-DLOOP variant (194) showed interactions with variants in genes of Complexes I, III, IV and V (*MT-ND4*, *MT-CYB*, *MT-COX3* and *MT-ATP8*, respectively); the joint presence of certain genotypes of these variants may affect the levels of TAU in CSF for individuals with MCI, increasing these levels, a trend seen for the investigated AD cases. As for the variant located in the Complex I gene, *MT-ND5*(13135), it presented interactions with variants in genes encoding Complexes I (one variant in *MT-ND2* and one in *MT-ND3*), III (two variants in *MT-CYB*) and IV (one variant in *MT-COX1*; notably, specific genotypes of *MT-COX1*(7476) and *MT-CYB*(15257) jointly with the GG genotype for *MT-ND5*(13135) may affect TAU levels in both cognitively healthy and AD individuals, and the same pattern was observed for *MT-ND3*(10172) in MCI patients. These findings reflect the intricate network of mtDNA epistasis in the progression of AD.

Currently, only a few studies are found in the global literature on mtDNA epistasis in diseases, and most of them focus on mitonuclear interactions, reinforcing the need for a closer look at this phenomenon specifically within the mitochondrial genome. For instance, a recent study by Duarte-Guterman et al. [57] found sex differences in hippocampal volume and greater memory decline in females compared to males, due to CSF tau-pathology being elevated in female carriers of APOEϵ4 in the ADNI cohort, corroborating our findings in the sex analysis for the mtDNA epistasis.

Furthermore, a study by Andrews et al. [58] investigated mitonuclear interactions in AD, analyzing associations between mtDNA haplogroups and nuclear-encoded mitochondrial genes for polygenic risk scores of this neurodegenerative disease, and reported both positive and negative epistatic interactions, indicating that epistasis between nuclear and mitochondrial genomes may influence the risk and the age of onset of AD. In this context, to the best of our knowledge, our study is the first to explore epistasis within the mitochondrial genome in AD.

### Limitations

We considered *p*≤ 0.05 as statistically significant after FDR correction, but we acknowledge the recent movement in the scientific community to lower this traditional threshold to 0.005. Thus, we encourage readers to take this into consideration when interpreting our results. In addition, future studies with an increased sample size and/or functional approaches are needed to further validate the associations suggested here.

## 5. Conclusions

In this exploratory study, by employing an integrative analysis with mitochondrial gene expression and genome-wide epistasis approaches, we identified differentially expressed genes in brain tissues from AD patients and epistatic interactions within the mitochondrial genome with a potential influence on the CSF levels of AD-related proteins, revealing important layers of interactions involving the mitochondrial genetics and molecular alterations with a potential impact on the development and progression of AD. Future investigations with larger cohorts or with functional approaches are encouraged to strengthen these findings.

## Figures and Tables

**Table 1:** RNA-seq experiments: number of samples (N) and age range in years.

GEO Accession	Brain Tissue	Group/Samples/Age	
GSE104704	Lateral temporal lobe	Healthy young adults	8/(42–59)
		Healthy elderly adults	10/(61–77)
		AD cases	12/(61–79)
GSE125583	Fusiform gyrus	Healthy elderly adults	70/(71–103)
		AD cases	219/(60–103)

**Table 2:** Statistical summary of tissue-specific differential expression analysis of mtDNA genes.

(1) Healthy Young Adults vs. AD Cases—GSE104704					
**HGNC Symbol**	**Gene**	**logFC**	**FDR**	**Expression**	**Biotype**
MT-TL1	ENSG00000209082.1	−1.84	0.0014	Down	tRNA
MT-TV	ENSG00000210077.1	−2.16	6.38 $\times \;10^{-5}$	Down	tRNA
MT-TM	ENSG00000210112.1	−1.93	0.0002	Down	tRNA
MT-TH	ENSG00000210176.1	−2.07	0.0025	Down	tRNA
MT-TS2	ENSG00000210184.1	−2.12	0.0018	Down	tRNA
MTND2P28	ENSG00000225630.1	−6.96	0.0014	Down	pseudogene
MTND1P23	ENSG00000225972.1	5.14	0.0002	Up	pseudogene
MTND1P20	ENSG00000226794.1	−2.04	0.0481	Down	pseudogene
MTND1P21	ENSG00000235940.1	−1.32	0.0151	Down	pseudogene
MTND5P11	ENSG00000248923.1	−2.52	0.0419	Down	pseudogene
MTND4P9	ENSG00000250050.1	−2.18	0.0302	Down	pseudogene
MTRNR2L1	ENSG00000256618.1	−6.92	0.0050	Down	protein coding
**(2) Health Elderly Adults vs. AD Cases—GSE104704**					
**HGNC Symbol**	**Gene**	**logFC**	**FDR**	**Expression**	**Biotype**
MTND1P23	ENSG00000225972.1	5.57	0.0001	Up	pseudogene
MTND2P12	ENSG00000228725.3	2.49	0.0158	Up	pseudogene
MTRNR2L1	ENSG00000256618.1	−4.19	0.0430	Down	protein coding
**(3) Health Elderly Adults vs. AD Cases—GSE125583**					
**HGNC Symbol**	**Gene**	**logFC**	**FDR**	**Expression**	**Biotype**
MTND1P23	ENSG00000225972.1	−2.13	1.36 $\times \;10^{-6}$	Down	pseudogene
MTND6P3	ENSG00000254132.1	−1.98	0.02653	Down	pseudogene
MTRNR2L2	ENSG00000271043.1	3.90	1.13 $\times \;10^{-56}$	Up	protein coding

## Data Availability

The data from the RNA-Seq experiments are publicly available at Gene Expression Omnibus under accession numbers GSE104704 and GSE125583. The mtDNA data were obtained from the ADNI database (https://adni.loni.usc.edu, accessed on 4 August 2021). The ADNI was launched in 2003 as a public–private partnership, led by Principal Investigator Michael W. Weiner, MD. The primary goal of ADNI has been to test whether serial magnetic resonance imaging (MRI), positron emission tomography (PET), other biological markers and clinical and neuropsychological assessment can be combined to measure the progression of mild cognitive impairment (MCI) and early Alzheimer’s disease (AD). The Principal Investigator of this initiative is Michael W. Weiner, MD, VA Medical Center and University of California—San Francisco. ADNI is a global research effort that actively supports the investigation and development of treatments that slow or stop the progression of AD and subjects have been recruited from over 50 sites across the US and Canada. The overall goal of ADNI is to determine biomarkers for use in Alzheimer’s disease clinical treatment trials. To date, it has three phases: ADNI-1, ADNI-GO and ADNI-2, consisting of cognitively normal (CN) individuals, early mild cognitive impairment (EMCI) to late mild cognitive impairment (LMCI) and dementia or AD. For more information, see http://www.adni-info.org, accessed on 4 August 2021. Institutional review board approval was conducted at each ADNI site. Written informed consent was obtained from all participants or authorized representative.

## References

[1] Oliver D., Reddy P.H. (2019). Molecular basis of Alzheimer’s disease: Focus on mitochondria. J. Alzheimer’s Dis..

[2] Bäckman L., Jones S., Berger A.K., Laukka E.J., Small B. (2004). Multiple cognitive deficits during the transition to Alzheimer’s disease. J. Intern. Med..

[3] Bistaffa E., Tagliavini F., Matteini P., Moda F. (2020). Contributions of Molecular and Optical Techniques to the Clinical Diagnosis of Alzheimer’s Disease. Brain Sci..

[4] (2020). 2020 Alzheimer’s disease facts and figures. Alzheimer’s Dement..

[5] Podlesniy P., Llorens F., Puigròs M., Serra N., Sepúlveda-Falla D., Schmidt C., Hermann P., Zerr I., Trullas R. (2020). Cerebrospinal Fluid Mitochondrial DNA in Rapid and Slow Progressive Forms of Alzheimer’s Disease. Int. J. Mol. Sci..

[6] Tanaka M., Török N., Vécsei L., Riederer P., Laux G., Nagatsu T., Le W., Riederer C. (2020). Novel Pharmaceutical Approaches in Dementia. NeuroPsychopharmacotherapy.

[7] Araújo G.S., Souza M.R., Oliveira J.R.M., Costa I.G. (2013). Random Forest and Gene Networks for Association of SNPs to Alzheimer’s Disease. Proceedings of the Brazilian Symposium on Bioinformatics.

[8] Souza M.B.R., Araújo G.S., Costa I.G., Oliveira J.R.M. (2016). Combined genome-wide CSF A*β*-42’s associations and simple network properties highlight new risk factors for Alzheimer’s disease. J. Mol. Neurosci..

[9] Brito L.M., Ribeiro-dos Santos Â., Vidal A.F., de Araújo G.S. (2020). Differential expression and mirna–gene interactions in early and late mild cognitive impairment. Biology.

[10] Cenini G., Voos W. (2019). Mitochondria as potential targets in Alzheimer disease therapy: An update. Front. Pharmacol..

[11] Delbarba A., Abate G., Prandelli C., Marziano M., Buizza L., Arce Varas N., Novelli A., Cuetos F., Martínez C., Lanni C. (2016). Mitochondrial alterations in peripheral mononuclear blood cells from Alzheimer’s disease and mild cognitive impairment patients. Oxid. Med. Cell. Longev..

[12] Zou T., Chen W., Zhou X., Duan Y., Ying X., Liu G., Zhu M., Pari A., Alimu K., Miao H. (2019). Association of multiple candidate genes with mild cognitive impairment in an elderly Chinese Uygur population in Xinjiang. Psychogeriatrics.

[13] Miton C.M., Buda K., Tokuriki N. (2021). Epistasis and intramolecular networks in protein evolution. Curr. Opin. Struct. Biol..

[14] Wang H., Bennett D.A., De Jager P.L., Zhang Q.Y., Zhang H.Y. (2021). Genome-wide epistasis analysis for Alzheimer’s disease and implications for genetic risk prediction. Alzheimer’s Res. Ther..

[15] Escott-Price V., Schmidt K.M. (2021). Probability of the Alzheimer’s disease based on common and rare genetic variants. Alzheimer’s Res. Ther..

[16] Tan M.S., Yang Y.X., Xu W., Wang H.F., Tan L., Zuo C.T., Dong Q., Tan L., Suckling J., Yu J.T. (2021). Associations of Alzheimer’s disease risk variants with gene expression, amyloidosis, tauopathy, and neurodegeneration. Alzheimer’s Res. Ther..

[17] Barrett T., Wilhite S.E., Ledoux P., Evangelista C., Kim I.F., Tomashevsky M., Marshall K.A., Phillippy K.H., Sherman P.M., Holko M. (2012). NCBI GEO: Archive for functional genomics data sets—Update. Nucleic Acids Res..

[18] Lonsdale J., Thomas J., Salvatore M., Phillips R., Lo E., Shad S., Hasz R., Walters G., Garcia F., Young N. (2013). The genotype-tissue expression (GTEx) project. Nat. Genet..

[19] Ridge P.G., Wadsworth M.E., Miller J.B., Saykin A.J., Green R.C., Kauwe J.S., The Alzheimer’s Disease Neuroimaging Initiative (2018). Assembly of 809 whole mitochondrial genomes with clinical, imaging, and fluid biomarker phenotyping. Alzheimer’s Dement..

[20] Lakatos A., Derbeneva O., Younes D., Keator D., Bakken T., Lvova M., Brandon M., Guffanti G., Reglodi D., Saykin A. (2010). Association between mitochondrial DNA variations and Alzheimer’s disease in the ADNI cohort. Neurobiol. Aging.

[21] Swerdlow R.H., Hui D., Chalise P., Sharma P., Wang X., Andrews S.J., Pa J., Mahnken J.D., Morris J., Wilkins H.M. (2020). Exploratory analysis of mtDNA haplogroups in two Alzheimer’s longitudinal cohorts. Alzheimer’s Dement..

[22] Trojanowski J.Q., Vandeerstichele H., Korecka M., Clark C.M., Aisen P.S., Petersen R.C., Blennow K., Soares H., Simon A., Lewczuk P. (2010). Update on the biomarker core of the Alzheimer’s Disease Neuroimaging Initiative subjects. Alzheimer’s Dement..

[23] Leinonen R., Sugawara H., Shumway M., International Nucleotide Sequence Database Collaboration (2010). The sequence read archive. Nucleic Acids Res..

[24] Gordon A., Hannon G. (2017). Fastx-Toolkit. FASTQ/A Short-Reads Pre-Processing Tools. http://hannonlab.cshl.edu/fastx_toolkit.

[25] Ewels P., Magnusson M., Lundin S., Käller M. (2016). MultiQC: Summarize analysis results for multiple tools and samples in a single report. Bioinformatics.

[26] Bolger A.M., Lohse M., Usadel B. (2014). Trimmomatic: A flexible trimmer for Illumina sequence data. Bioinformatics.

[27] Dobin A., Davis C.A., Schlesinger F., Drenkow J., Zaleski C., Jha S., Batut P., Chaisson M., Gingeras T.R. (2013). STAR: Ultrafast universal RNA-seq aligner. Bioinformatics.

[28] Anders S., Pyl P.T., Huber W. (2015). HTSeq—A Python framework to work with high-throughput sequencing data. Bioinformatics.

[29] Robinson M.D., McCarthy D.J., Smyth G.K. (2010). edgeR: A Bioconductor package for differential expression analysis of digital gene expression data. Bioinformatics.

[30] Warde-Farley D., Donaldson S.L., Comes O., Zuberi K., Badrawi R., Chao P., Franz M., Grouios C., Kazi F., Lopes C.T. (2010). The GeneMANIA prediction server: Biological network integration for gene prioritization and predicting gene function. Nucleic Acids Res..

[31] Purcell S., Neale B., Todd-Brown K., Thomas L., Ferreira M.A., Bender D., Maller J., Sklar P., De Bakker P.I., Daly M.J. (2007). PLINK: A tool set for whole-genome association and population-based linkage analyses. Am. J. Hum. Genet..

[32] Cavalcante G.C., Magalhães L., Ribeiro-dos Santos Â., Vidal A.F. (2020). Mitochondrial Epigenetics: Non-Coding RNAs as a Novel Layer of Complexity. Int. J. Mol. Sci..

[33] Gammage P.A., Frezza C. (2019). Mitochondrial DNA: The overlooked oncogenome?. BMC Biol..

[34] Streck E.L., Gonçalves C.L., Furlanetto C.B., Scaini G., Dal-Pizzol F., Quevedo J. (2014). Mitochondria and the central nervous system: Searching for a pathophysiological basis of psychiatric disorders. Braz. J. Psychiatry.

[35] Cabral-Costa J., Kowaltowski A. (2020). Neurological disorders and mitochondria. Mol. Asp. Med..

[36] Swerdlow R.H., Khan S.M. (2004). A “mitochondrial cascade hypothesis” for sporadic Alzheimer’s disease. Med. Hypotheses.

[37] Swerdlow R.H., Burns J.M., Khan S.M. (2010). The Alzheimer’s disease mitochondrial cascade hypothesis. J. Alzheimer’s Dis..

[38] Ridge P.G., Kauwe J.S. (2018). Mitochondria and Alzheimer’s disease: The role of mitochondrial genetic variation. Curr. Genet. Med. Rep..

[39] Compagnoni G.M., Di Fonzo A., Corti S., Comi G.P., Bresolin N., Masliah E. (2020). The Role of Mitochondria in Neurodegenerative Diseases: The Lesson from Alzheimer’s Disease and Parkinson’s Disease. Mol. Neurobiol..

[40] Halliday G. (2017). Pathology and hippocampal atrophy in Alzheimer’s disease. Lancet Neurol..

[41] Pierrick C., Manjón J.V., Enrique L., Gwenaelle C. (2019). Lifespan Changes of the Human Brain In Alzheimer’s Disease. Sci. Rep..

[42] Whitwell J.L. (2010). Progression of atrophy in Alzheimer’s disease and related disorders. Neurotox. Res..

[43] Sintini I., Graff-Radford J., Senjem M.L., Schwarz C.G., Machulda M.M., Martin P.R., Jones D.T., Boeve B.F., Knopman D.S., Kantarci K. (2020). Longitudinal neuroimaging biomarkers differ across Alzheimer’s disease phenotypes. Brain.

[44] Kiernan J. (2012). Anatomy of the temporal lobe. Epilepsy Res. Treat..

[45] Goldstein I.S., Erickson D.J., Sleeper L.A., Haynes R.L., Kinney H.C. (2017). The lateral temporal lobe in early human life. J. Neuropathol. Exp. Neurol..

[46] Palejwala A.H., O’Connor K.P., Milton C.K., Anderson C., Pelargos P., Briggs R.G., Conner A.K., O’Donoghue D.L., Glenn C.A., Sughrue M.E. (2020). Anatomy and white matter connections of the fusiform gyrus. Sci. Rep..

[47] Weiner K.S., Zilles K. (2016). The anatomical and functional specialization of the fusiform gyrus. Neuropsychologia.

[48] Tak S.H., Hong S.H. (2014). Face-name memory in Alzheimer’s disease. Geriatr. Nurs..

[49] Salinas-Giegé T., Giegé R., Giegé P. (2015). tRNA biology in mitochondria. Int. J. Mol. Sci..

[50] Garone C., Minczuk M., D’Souza A.R. (2018). Mitochondrial transcription and translation: Overview. Essays Biochem..

[51] Bik-Multanowski M., Pietrzyk J.J., Midro A. (2015). MTRNR2L12: A candidate blood marker of early Alzheimer’s disease-like dementia in adults with down syndrome. J. Alzheimer’s Dis..

[52] Hashimoto Y., Niikura T., Tajima H., Yasukawa T., Sudo H., Ito Y., Kita Y., Kawasumi M., Kouyama K., Doyu M. (2001). A rescue factor abolishing neuronal cell death by a wide spectrum of familial Alzheimer’s disease genes and A*β*. Proc. Natl. Acad. Sci. USA.

[53] Gong Z., Tas E., Muzumdar R. (2014). Humanin and age-related diseases: A new link?. Front. Endocrinol..

[54] Bodzioch M., Lapicka-Bodzioch K., Zapala B., Kamysz W., Kiec-Wilk B., Dembinska-Kiec A. (2009). Evidence for potential functionality of nuclearly-encoded humanin isoforms. Genomics.

[55] Moss D.J.H., Pardiñas A.F., Langbehn D., Lo K., Leavitt B.R., Roos R., Durr A., Mead S., Coleman A., Santos R.D. (2017). Identification of genetic variants associated with Huntington’s disease progression: A genome-wide association study. Lancet Neurol..

[56] Hazafa A., Batool A., Ahmad S., Amjad M., Chaudhry S.N., Asad J., Ghuman H.F., Khan H.M., Naeem M., Ghani U. (2021). Humanin: A mitochondrial-derived peptide in the treatment of apoptosis-related diseases. Life Sci..

[57] Duarte-Guterman P., Albert A.Y., Barha C.K., Galea L.A.M., on behalf of the Alzheimer’s Disease Neuroimaging Initiative (2021). Sex influences the effects of APOE genotype and Alzheimer’s diagnosis on neuropathology and memory. Psychoneuroendocrinology.

[58] Andrews S.J., Fulton-Howard B., Patterson C., McFall G.P., Gross A., Michaelis E.K., Goate A., Swerdlow R.H., Pa J., Initiative A.D.N. (2020). Mitonuclear interactions influence Alzheimer’s disease risk. Neurobiol. Aging.

